# Kinetics of Boron Recovery from Boron-Rich Slag via Low-Temperature Soda Roasting

**DOI:** 10.3390/ma19030469

**Published:** 2026-01-24

**Authors:** Jie Li, Jinbiao Li, Guolu Lv, Yanfen Li, Yan Lu, Zhaoxin Du, Quhan Mu

**Affiliations:** 1 School of Materials Science and Engineering, Inner Mongolia University of Technology, Hohhot 010051, China; ljws@imut.edu.cn (J.L.); liyf980320@imut.edu.cn (Y.L.); luyan_qq@sina.com (Y.L.); duzhaoxin@163.com (Z.D.); 2 Process Technology Department, Hubei EVE Energy Co., Ltd., Jingmen 448000, China; lihs777@163.com; 3 SPIC Inner Mongolia Baiyinhua Aluminum & Power Co., Ltd., Xilingol 026000, China; 15147040681@163.com

**Keywords:** boron-rich slag, low-temperature soda roasting, boron leaching rate, kinetics, apparent activation energy

## Abstract

This study proposes an innovative process of low-temperature soda roasting followed by water leaching to extract boron and produce borax from boron-rich slag. To further enhance the leaching rate of boron, pretreatment of the boron-rich slag with the nucleating agent TiO_2_ was conducted. The effects of roasting temperature and Na_2_CO_3_ addition on the boron leaching rate, as well as the roasting kinetics of the TiO_2_-nucleated furnace-cooled slag, were investigated. The results indicate that at a roasting temperature of 700 °C for 150 min, the maximum boron leaching rate can reach 88.65%. The reaction of low-temperature soda roasting for TiO_2_-nucleated furnace-cooled slag to produce Na_2_B_6_O_10_ is controlled by interfacial chemical reaction, with an apparent activation energy of 88.677 kJ/mol.

## 1. Introduction

Boron, a critical strategic element, plays an indispensable role across industrial, agricultural, and national defense sectors. With the continuous expansion of the global economy in the 21st century, the market demand for boron-containing compounds has been growing substantially [1,2,3,4]. Naturally occurring boron is primarily found in the form of minerals such as ascharite and ludwigite, with significant deposits located in China, Turkey, Russia, and the United States. In China, the depletion of high-quality ascharite reserves and the rising domestic demand have established ludwigite as a key substitute feedstock, which is now becoming the primary raw material for the boron industry [5,6,7]. For the exploitation and utilization of ludwigite, China has formulated a guiding principle centered on “Boron-focused, Comprehensive Utilization, and Environmental Protection” [8]. In this context, the pyrometallurgical process is widely regarded as the optimal pathway, as it enables the efficient extraction of boron while simultaneously achieving high utilization rates of iron and magnesium. This process involves smelting ludwigite in a blast furnace to produce two primary products: boron-containing pig iron and boron-rich slag.

Boron-rich slag is a type of boron-containing secondary resource with significant recovery value [9,10,11]. The B_2_O_3_ content in boron-rich slag can reach up to 17%, and the boron primarily exists in an amorphous glassy state [12,13,14,15,16]. Currently, the primary methods for recovering boron from boron-rich slag include the sulfuric acid method, the CO_2_-soda method, and the molten boron-rich slag sodification method. The sulfuric acid method [17,18,19] is hindered by significant drawbacks, including high acid consumption, severe equipment corrosion, and environmental pollution concerns. The CO_2_-soda method [20,21] requires the boron in the raw material to exhibit high reactivity. However, the reactivity of boron in boron-rich slag is relatively low [22]. The molten boron-rich slag sodification method [23,24] is plagued by significant drawbacks, including substantial loss of Na_2_CO_3_ and difficulties in controlling its dosage. The method of low-temperature soda roasting combined with water leaching offers an innovative route for borax preparation by enabling efficient boron extraction from boron-rich slag [25,26]. This process mainly involves two stages: first, roasting the boron-rich slag with Na_2_CO_3_ at low temperatures, followed by water leaching of the roasted product. During roasting, boron compounds in the slag are converted into water-soluble sodium borates, which are subsequently dissolved into the solution during the leaching step. Borax is finally obtained through concentration and crystallization of the leach solution. To enhance the boron leaching efficiency from roasted slag, current research emphasizes optimization of the low-temperature soda roasting process and investigation of its kinetics.

To address these challenges, this study systematically investigated the boron recovery process from boron-rich slag via low-temperature soda roasting and water leaching. Specifically, the slag was pretreated with TiO_2_ as a nucleating agent, roasted with Na_2_CO_3_ at varying temperatures and additive amounts, and then water-leached. The effects of key parameters were evaluated, and the kinetics of the roasting reaction were analyzed to determine the rate-controlling step and apparent activation energy.

## 2. Materials and Methods

### 2.1. Experimental Raw Materials

The boron-rich slag sample, sourced from Yingkou Guangda Industrial Co., Ltd. (Yingkou, China), had its chemical composition detailed in Table 1. Its B_2_O_3_ content was determined by titration with standard NaOH solution [27], and other oxides were quantified by X-ray fluorescence (XRF) spectrometry.

**Figure 1:**
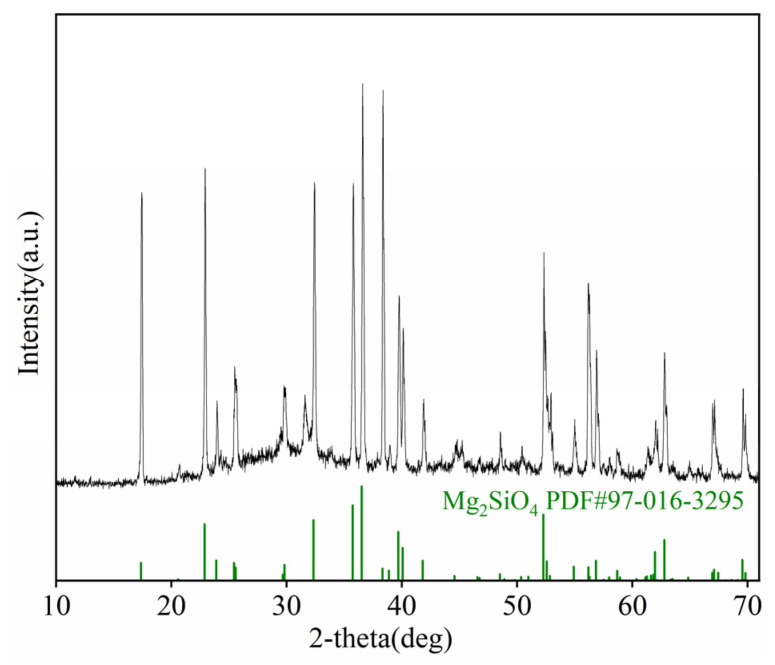
XRD pattern of the boron-rich slag.

As shown in Table 1, the slag contains 11.38% B_2_O_3_, along with 32.73% MgO and 17.28% CaO. The mineralogical composition was analyzed using X-ray diffraction (XRD) performed on a Rigaku D/max 2500/PC diffractometer (Rigaku Corporation, Tokyo, Japan) with Cu-Kα radiation and a step size of 0.02°. The XRD pattern (Figure 1) indicates that magnesium olivine (Mg_2_SiO_4_) is the dominant crystalline phase. The few minor, low-intensity peaks are attributable to trace crystalline components within the predominantly amorphous slag matrix. The boron is predominantly present in an amorphous glass phase within the slag matrix, as evidenced by the absence of crystalline boron-containing phases in the XRD patterns.

### 2.2. Experimental Methods

The boron-rich slag was sieved to under 74 μm (200 mesh). Subsequently, 49.5 g of the sieved slag was thoroughly mixed with 0.5 g of analytically pure TiO_2_ in a ball mill. To prevent agglomeration, 1–2 drops of anhydrous ethanol were added during the mixing process. The mixing was conducted at a rotational speed of 240 r/min for a duration of 15 min. The homogenized powder was charged into a graphite crucible and subsequently transferred to a silicon-molybdenum bar muffle furnace for subsequent processing. The furnace was heated to 1450 °C at a rate of 10 °C/min under an air atmosphere, maintained at this temperature for 10 min, and then furnace-cooled to room temperature. The resulting product was designated as TiO_2_-nucleated furnace-cooled slag. This product was ground using a sample pulverizer to a particle size of less than 74 μm for subsequent roasting experiments.

15 g of the TiO_2_ furnace-cooled slag was weighed, and analytical pure Na_2_CO_3_ was added according to a specified molar ratio based on the B_2_O_3_ content in the slag. The entire mixture was loaded into a ball mill, followed by the addition of 1–2 drops of anhydrous ethanol. Blending was performed at 240 r/min for 15 min to achieve thorough homogenization. Subsequently, 15 g of the homogenized mixture was weighed and transferred into a corundum crucible. The crucible was then placed in a resistance furnace preheated to 700 °C for a low-temperature roasting process lasting 2 h. After roasting, the crucible was furnace-cooled to room temperature. The resulting product was denoted as Na_2_CO_3_-modifed slag (NMS).

NMS (10 g) was charged into a single-neck flask. Deionized water (100 mL) was added at a liquid-to-solid mass ratio of 10:1, followed by the introduction of several boiling chips to prevent bumping during the leaching process. The mixture was subjected to water leaching under boiling reflux conditions for 2 h. The resultant leachate was separated via a Buchner funnel, and the filter cake was rinsed three times with hot deionized water. The filter cake was then dried at 120 °C to constant weight and weighed.

The boron leaching rate from the boron-rich slag was calculated using Equation (1), with the B_2_O_3_ content in both the NMS and the leached residue being determined by titration against a standard NaOH solution [27].

(1) $$\eta_{B}=\frac{m_{\mathrm{NMS}}{\;\times \;w}_{\mathrm{NMS}}-m_{\mathrm{lr}}{\;\times \;w}_{\mathrm{lr}}}{m_{\mathrm{NMS}}\;\times {\;w}_{\mathrm{NMS}}}$$

Here, *m_NMS_* and *w_NMS_* are the mass and B_2_O_3_ content of the NMS, and *m_lr_* and *w_l_*_r_ are those of the leached residue, respectively.

### 2.3. Experimental Principle

**Figure 2:**
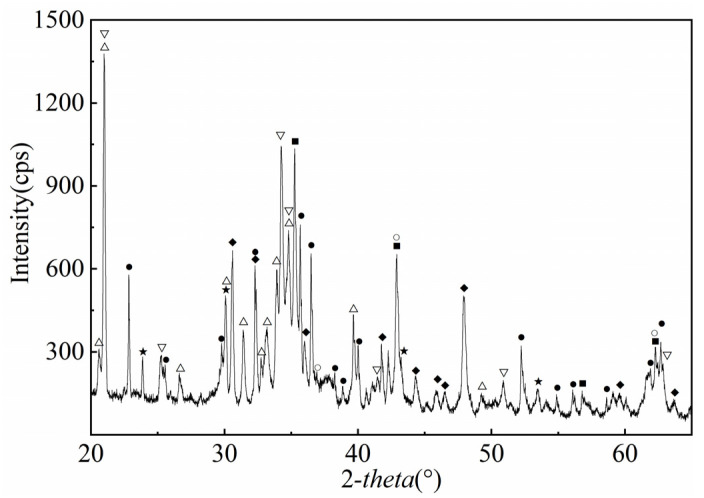
XRD pattern of NMS. ● Mg_2_SiO_4_; ○ MgO; ◆ Ca_3_B_2_O_6_; △ Na_2_MgSiO_4_; ▽ Na_3_MgAlSi_2_O_8_; ★ Na_2_B_6_O_10_; ■ Mg_2_TiO_4._

Figure 2 shows the XRD pattern of the NMS. As observed in Figure 2, the boron-containing crystalline phases in the slag primarily consist of Na_2_B_6_O_10_ and Ca_3_B_2_O_6_. At temperatures between 600 and 700 °C, the boron-rich slag reacts with Na_2_CO_3_ during low-temperature roasting, where Na_2_CO_3_ can interact with the boron components in the amorphous glassy phase to form Na_2_B_6_O_10_. The resulting Na_2_B_6_O_10_ subsequently crystallizes into the Na_2_B_6_O_10_ phase under the action of the nucleating agent TiO_2_. Additionally, a side reaction occurs during roasting between calcium and boron components, yielding Ca_3_B_2_O_6_ as a product. Ca_3_B_2_O_6_ is insoluble in water [29], whereas Na_2_B_6_O_10_ is readily soluble. Through water leaching of the NMS, Na_2_B_6_O_10_ can be transferred into the solution. Following the water leaching step, borax can be prepared through the subsequent concentration and crystallization of the obtained aqueous solution. The Na_2_MgSiO_4_, MgO, and Mg_2_TiO_4_ phases exist in the NMS; these phases are the reaction products of NMS with Na_2_CO_3_ and may undergo the reactions shown in Equations (2) and (3) [30,31].

(2) $${Na}_{2}{CO}_{3}+{Mg}_{2}{SiO}_{4}={Na}_{2}{MgSiO}_{4}+MgO+CO_{2}$$

(3) $$2MgO+TiO_{2}={Mg}_{2}TiO_{4}$$

## 3. Results and Discussion

### 3.1. Kinetics of Low-Temperature Soda Roasting of TiO_2_-Nucleated Furnace-Cooled Slag

#### 3.1.1. The Effect of Roasting Temperature on the Boron Leaching Rate

**Figure 3:**
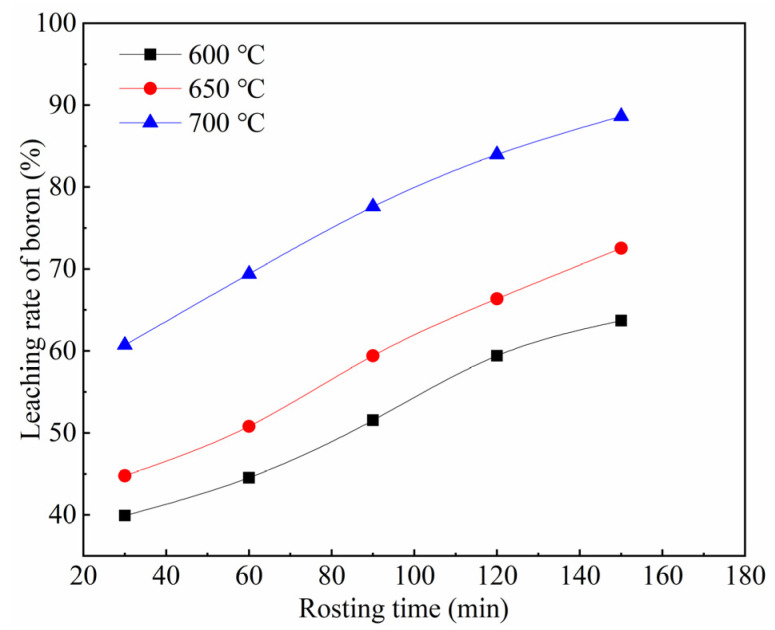
Boron leaching rate versus roasting temperature.

The influence of roasting temperature on the boron leaching rate was investigated with a Na_2_CO_3_ dosage of 4 times the theoretical amount (based on a 1:1 molar ratio of Na_2_CO_3_ to B_2_O_3_) and a liquid-to-solid ratio of 10:1, under 2 h of boiling reflux leaching. The experimental results are shown in Figure 3.

As can be seen from Figure 3, the roasting temperature has a significant influence on the boron leaching rate from the TiO_2_-nucleated furnace-cooled slag. The boron leaching rate exhibits a positive correlation with the roasting temperature over the same time period. At a roasting time of 150 min, raising the temperature from 600 °C to 700 °C increased the boron leaching rate from 63.72% to 88.65%. This enhancement is due to the elevated temperature, which increases the solid-phase diffusion coefficient, accelerates the interfacial reaction, and promotes the nucleation and growth of Na_2_B_6_O_10_ [32].

#### 3.1.2. Reaction Control Steps

The low-temperature soda roasting of boron-rich slag constitutes a solid–solid reaction [33], with three potential rate-controlling steps: chemical reaction control at the interface between reactant and product phases, nucleation and growth control during the reaction process, and diffusion control [34,35]. In solid-state reaction models, there are typically interfacial reaction-controlled models, nucleation-controlled models, and diffusion-controlled models, each with its unique kinetic model functions. The typical kinetic equations for the three types of solid-state reactions are shown in Equations (4)–(7). If the reaction during the low-temperature soda roasting of boron-rich slag is controlled by interfacial chemical reaction and follows a zero-order reaction, the kinetic model can be expressed as [33,36]

(4) $$1\;-\;{(1\;-\;x)}^{1/3}=\mathrm{kt}$$

When the reaction follows first-order kinetics and the change in reaction interface area cannot be neglected, the kinetic model can be expressed as [33,36]

(5) $${(1\;-\;x)}^{-2\text{∕}3}\;-\;1=\mathrm{kt}$$

For a reaction controlled by nucleation and growth during low-temperature soda roasting, the kinetic model is expressed as [37,38]

(6) $$-\ln\left(1\;-\;x\right)=\mathrm{kt}$$

For a diffusion-controlled reaction during low-temperature soda roasting, the kinetic model can be expressed as [39,40]

(7) $$1\;-\;\frac{2}{3}x\;-\;{\left(1\;-\;x\right)}^{2/3}=\mathrm{kt}$$

In these equations, *x* represents the boron leaching rate from the boron-rich slag, %; *t* denotes the roasting time, min; and *k* is the reaction rate constant, min^−1^.

**Figure 4:**
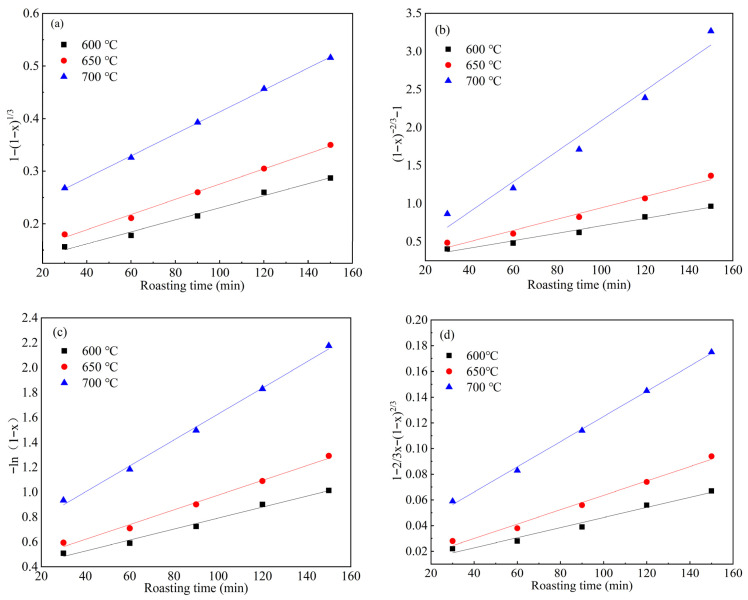
Linear fitting curves of kinetic equations at different temperatures. (**a**) $1\;-\;{(1\;-\;x)}^{1/3}$ vs. *t*; (**b**) ${(1\;-\;x)}^{-2\text{∕}3}\;-\;1$ vs. *t*; (**c**) $-\ln\left(1\;-\;x\right)$ vs. *t*; (**d**) $1\;-\;\frac{2}{3}x\;-\;{\left(1\;-\;x\right)}^{2/3}$ vs. *t*.

The experimental data from Figure 3 were substituted into Equations (4)–(7), respectively, and linear regression analysis was performed using the least squares method. The results are shown in Figure 4. As can be seen from Figure 4a, the relationship between 1 − (1 − *x*)^1/3^ and time t exhibits a good fit, with the experimental data closely distributed around the fitted straight line. Table 2 presents the linear fitting coefficients of determination (R^2^) and rate constants (*k*) for the different kinetic equations at various roasting temperatures. The average R^2^ value for Equation (4) across the three reaction temperatures was 0.9920, which is the highest and closest to 1, indicating the best fitting quality for Equation (4). Therefore, the optimal fit is observed between 1 − (1 − *x*)^1/3^ and time *t*, suggesting that the soda roasting reaction of TiO_2_-nucleated furnace-cooled slag to form Na_2_B_6_O_10_ at low temperatures is controlled by the interface chemical reaction.

#### 3.1.3. Calculation of the Apparent Activation Energy

The Arrhenius equation (Equation (8)), which describes the dependence of the chemical reaction rate constant on temperature, can be employed to calculate the apparent activation energy of the reaction.

(8) $$k\;={\;\mathrm{Ae}}^{-\frac{E_{a}}{\mathrm{RT}}}$$

Taking the logarithm of both sides of Equation (8) yields Equation (9):

(9) $$\mathrm{lnk}\;=\;-\frac{E_{a}}{\mathrm{RT}}\;+\;\mathrm{lnA}$$

where *k* is the reaction rate constant (min^−1^), *E_a_* is the apparent activation energy (J/mol), *T* is the temperature (K), *A* is the pre-exponential factor, and *R* is the gas constant (8.314 J·K^−1^·mol^−1^).

**Figure 5:**
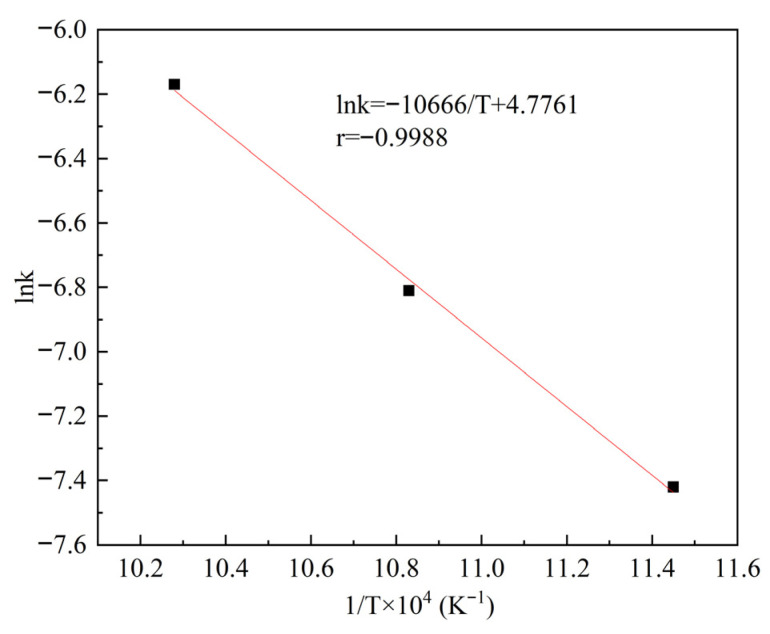
The relationship between ln*k* and 1/*T* for the low-temperature soda roasting reaction of TiO_2_ furnace-cooled slag.

The Arrhenius plot (lnk vs. 1/T) derived from the rate constants in Table 2 (Equation (4)) is presented in Figure 5. A linear fit reveals a strong correlation, from which the apparent activation energy was determined to be 88.677 kJ/mol using Equation (9). The pre-exponential factor (A = 118.64) was obtained from the intercept of the fitted line. Thus, the kinetic equation for the roasting process is expressed as

(10) $$1\;-\;{\left(1\;-\;x\right)}^{\frac{1}{3}}=118.64e^{-\frac{88677}{\mathrm{RT}}}t$$

According to previous research [28], in the absence of TiO_2_, the product was NaBO_2_, and the kinetics shifted from diffusion control to nucleation control with increasing Na_2_CO_3_ dosage. In contrast, with the addition of TiO_2_, the product becomes Na_2_B_6_O_10_, and the kinetics remain consistently interfacial chemical reaction-controlled across the entire temperature range. The apparent activation energy for the non-nucleated system was 54.45 kJ/mol, which increased significantly to 88.68 kJ/mol with TiO_2_ addition. This notable increase indicates that TiO_2_ alters the reaction pathway, most likely by facilitating the formation of a more complex, polymerized borate structure ([B_6_O_10_]^2−^). This structure possesses a higher intrinsic energy barrier for its interfacial formation step. Therefore, TiO_2_ acts not merely as a physical accelerator but as a mechanism-modifying agent that promotes a distinct and more structurally ordered crystallization pathway.

### 3.2. Effect of Na_2_CO_3_ Addition Amount on Boron Leaching Rate

**Figure 6:**
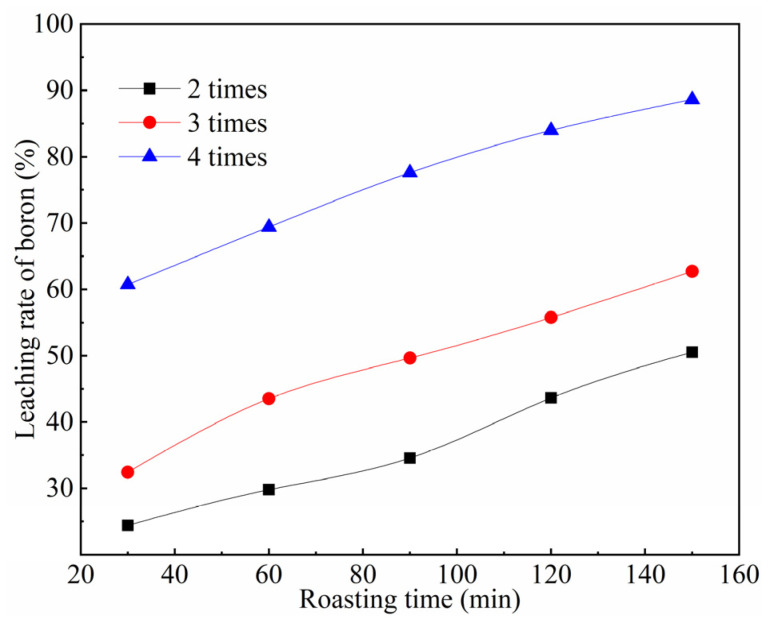
The effect of sodium carbonate addition on boron leaching rate.

The effect of Na_2_CO_3_ addition on the boron leaching rate was investigated under fixed conditions of a roasting temperature of 700 °C, a liquid-to-solid ratio of 10:1, and a reflux leaching time of 2 h. The results are shown in Figure 6.

As illustrated in Figure 6, the amount of Na_2_CO_3_ added exerts a significant influence on the boron leaching rate from the TiO_2_-nucleated furnace-cooled slag. The boron leaching rate exhibits a positive correlation with the amount of Na_2_CO_3_ added over the same duration. Specifically, when the roasting time was 150 min, increasing the Na_2_CO_3_ addition amount from 2 to 4 times the theoretical amount resulted in a marked enhancement of the boron leaching rate from 50.53% to 88.65%. This improvement is attributed to the increased contact area between the slag particles and Na_2_CO_3_ at higher dosages, leading to a more complete reaction during roasting and thereby facilitating boron leaching.

**Figure 7:**
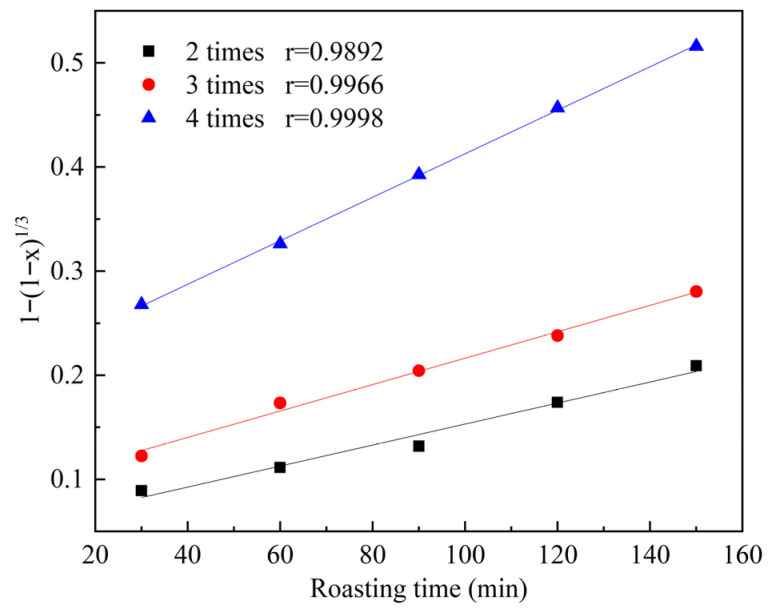
The relationship between 1 − (1 − x)^1/3^ and time at different amounts of sodium carbonate addition.

The data from Figure 6 were substituted into Equation (4) and subjected to linear fitting, with the resulting plot presented in Figure 7. As shown in Figure 7, the linear fitting correlation coefficients (*R^2^*) for Na_2_CO_3_ addition amounts of 2, 3, and 4 times the theoretical amount are 0.9892, 0.9966, and 0.9998, respectively. The strong linearity of the plot for Equation (4) confirms that, within the studied extent and timeframe, the roasting reaction is predominantly controlled by an interfacial chemical reaction mechanism.

## 4. Conclusions

The pre-treatment of boron-rich slag with TiO_2_ as a nucleating agent, followed by roasting with the addition of four times the theoretical amount of Na_2_CO_3_ at 700 °C for 150 min, and subsequent water leaching with a liquid-to-solid ratio of 10:1 under boiling reflux for 2 h, achieved a maximum boron leaching rate of 88.65%.

Within the temperature range of 600–700 °C, the soda roasting reaction of the TiO_2_-nucleated furnace-cooled slag to form Na_2_B_6_O_10_ is controlled by an interfacial chemical reaction mechanism. For this temperature range, the apparent activation energy for this process was determined to be 88.677 kJ/mol, and the kinetic equation is described by $1\;-\;{\left(1-\;x\right)}^{\frac{1}{3}}\;=\;118.64e^{-\frac{88677}{\mathrm{RT}}}t$. 

## Figures and Tables

**Table 1:** Chemical composition of the boron-rich slag [28].

Composition	B_2_O_3_	MgO	SiO_2_	CaO	Al_2_O_3_	S	Total Fe	FeO
Mass fraction, %	11.38	32.73	28.95	17.28	7.14	0.53	0.91	0.59

**Table 2:** *R*^2^ and *k* for different kinetic models at each temperature.

Model Equations	600 °C		650 °C		700 °C		Average of *R*^2^	Rank
	*k*	*R* ^2^	*k*	*R* ^2^	*k*	*R* ^2^		
Equation (4)	0.0006	0.9847	0.0011	0.9941	0.0021	0.9972	0.9920	1
Equation (5)	0.0049	0.9730	0.0074	0.9714	0.0200	0.9600	0.9681	4
Equation (6)	0.0044	0.9826	0.0059	0.9894	0.0104	0.9952	0.9891	2
Equation (7)	0.0004	0.9693	0.0006	0.9843	0.0010	0.9974	0.9837	3

## Data Availability

The original contributions presented in this study are included in the article. Further inquiries can be directed to the corresponding author.
